# Sex and survival following pulmonary endarterectomy for chronic thromboembolic pulmonary hypertension: a Scandinavian observational cohort study

**DOI:** 10.1177/20458940211056014

**Published:** 2021-10-27

**Authors:** Janica Kallonen, Kasper Korsholm, Fredrik Bredin, Matthias Corbascio, Mads Jønsson Andersen, Lars Bo Ilkjær, Søren Mellemkjær, Ulrik Sartipy

**Affiliations:** 1 Department of Molecular Medicine and Surgery, 27106 Karolinska Institutet, Stockholm, Sweden; 2 Department of Cardiothoracic Surgery, 59562 Karolinska University Hospital, Stockholm, Sweden; 3 Department of Cardiology, 11297 Aarhus University Hospital, Aarhus, Denmark; 4 Division of Perioperative Medicine and Intensive Care, Section Cardiothoracic Surgery and Anesthesiology, Karolinska University Hospital, Stockholm, Sweden; 5 Department of Cardiothoracic Surgery, 53146 Rigshospitalet, Rigshospitalet, Copenhagen, Denmark; 6 Department of Cardiothoracic Surgery, Aarhus University Hospital, Aarhus, Denmark

**Keywords:** sex-specific survival, life expectancy, epidemiology

## Abstract

Studies have suggested sex-related survival differences in chronic thromboembolic pulmonary hypertension (CTEPH). Whether long-term prognosis differs between men and women following pulmonary endarterectomy for CTEPH remains unclear. We investigated sex-specific survival after pulmonary endarterectomy for CTEPH. We included all patients who underwent pulmonary endarterectomy for CTEPH at two Scandinavian centers and obtained baseline characteristics and vital statuses from patient charts and national health-data registers. Propensity scores and weighting were used to account for baseline differences. Flexible parametric survival models were employed to estimate the association between sex and all-cause mortality and the absolute survival differences. The expected survival in an age-, sex-, and year of surgery matched general population was obtained from the Human Mortality Database, and the relative survival was used to estimate cause-specific mortality. A total of 444 patients were included, comprising 260 (59%) men and 184 (41%) women. Unadjusted 30-day mortality was 4.2% in men versus 9.8% in women (p = 0.020). In weighted analyses, long-term survival did not differ significantly in women compared with men (hazard ratio: 1.36; 95% confidence interval: 0.89–2.06). Relative survival at 15 years conditional on 30-day survival was 94% (79%–107%) in men versus 75% (59%–88%) in women. In patients who underwent pulmonary endarterectomy for CTEPH, early mortality was higher in women compared with men. After adjustment for differences in baseline characteristics, female sex was not associated with long-term survival. However, relative survival analyses suggested that the observed survival in men was close to the expected survival in the matched general population, whereas survival in women deviated notably from the matched general population.

Chronic thromboembolic pulmonary hypertension (CTEPH) is a potentially curable form of pulmonary hypertension.^1^,^2^ The gold standard treatment for operable CTEPH is major cardiac surgery with pulmonary endarterectomy (PEA) according to guidelines from the European Society of Cardiology.^3^ Inoperable patients are evaluated for medical treatment and balloon pulmonary angioplasty.^1,3^ Previous studies reported 10-year survival rates after PEA of 62%–75% in patients with CTEPH.^4–7^

In recent years, focus on sex differences in cardiopulmonary diseases has increased.^8^ In acute myocardial infarction, for example, the pathophysiology and outcome differ between the sexes according to a report from the American Heart Association.^9^ In CTEPH, little is known about how sex affects symptoms, hemodynamic parameters, treatment course, and ultimately prognosis. Previously, CTEPH was thought to affect both sexes equally.^10^

Using data from the European CTEPH registry, Barco et al.^11^ reported sex-specific differences in the clinical presentation of CTEPH, performance of PEA, and survival. Women with CTEPH had better overall long-term survival than men, even though women less frequently underwent PEA. Women also had fewer cardiovascular comorbidities requiring concomitant surgical treatment. The reason why women underwent PEA less frequently remained unclear, but the phenomenon was more pronounced in low-volume surgical centers.

To our knowledge, no studies have examined sex-specific survival after PEA. To investigate the differences in survival between the sexes after PEA, we performed a binational Scandinavian observational cohort study. The aim of the study was to determine sex-specific survival after PEA in Scandinavia.

## Methods

### Study design

This observational cohort study followed the Strengthening the Reporting of Observational Studies in Epidemiology (STROBE) guidelines for observational studies.^12^ Approval from the Swedish Ethical Review Authority was obtained and the need for informed consent was waived (registration numbers 2018/1296-31 and 2020-03130). The Central Denmark Region approved the study according to the Danish Health Act paragraph 42, section 2.

### Study population and data sources

The Swedish cohort included all patients who underwent PEA for CTEPH at Karolinska University Hospital between 1992 and 2020. In Sweden, most patients who underwent PEA during the study period were operated at Karolinska University Hospital, but a small number of patients were operated at another center and were excluded from the study. We obtained baseline characteristics and vital statuses from patient charts and national health-data registries using the Swedish personal identity numbers of the patients.^13,14^

The Danish cohort included all patients who underwent PEA for CTEPH between 1994 and 2020 at Aarhus University Hospital, which is the Danish National Center for PEA and the only center in Denmark that performs this procedure. Baseline characteristics were obtained from patient charts and vital statuses were obtained for all patients through the Danish Civil Registration System.^15^

Clinical results were previously published for subsets of the Danish^5,16^ and Swedish^7^ cohorts that included 50, 239, and 100 patients, respectively.

### Outcomes

The primary outcome measure was all-cause mortality. Person-time in days was calculated from the date of surgery until date of death or end of follow-up (6 May 2021 in the Swedish cohort and 16 November 2020 or 1 April 2021 in the Danish cohort).

### Statistical methods

Baseline characteristics were described as frequencies and percentages for categorical variables and means and standard deviations (SDs) for continuous variables. To address confounding (differences in measured baseline covariates between men and women), we estimated covariate balancing propensity scores (probability of female sex based on observed baseline characteristics) and calculated stabilized inverse probability of treatment weights for average treatment effects.^17^ The model included all of the variables shown in Table 1. Balance was assessed after weighting by standardized mean differences. Absolute standardized difference ≤0.1 was considered an ideal balance.^18^ In the weighted population, flexible parametric survival models were used to estimate survival as well as the absolute survival difference with 95% confidence interval (CI) between men and women at specified time points.^19^ Flexible parametric survival models were used to estimate the association between female sex and survival (with male sex as the reference category) expressed as the hazard ratio (HR) and 95% CI before and after weighting.

**Table 1.** Baseline characteristics in the 444 patients who underwent pulmonary endarterectomy in Sweden and Denmark between 1992 and 2020.

Variable	Total population	Men	Women	*p*-value	Missing data (%)
Number of patients	444	260 (58.6)	184 (41.4)		0
Center					
Denmark	324 (73.0)	185 (71.2)	139 (75.5)	0.359	0
Sweden	120 (27.0)	75 (28.8)	45 (24.5)		
Age (years), mean (SD)	60.7 (13.1)	61.8 (12.2)	59.2 (14.2)	0.039	0
Body mass index (kg/m^2^)				0.362	29.1
<18.5	4 (1.3)	2 (1.0)	2 (1.7)		
18.5–24.99	119 (37.8)	74 (37.6)	45 (38.1)		
25–29.9	123 (39.0)	83 (42.1)	40 (33.9)		
≥30	69 (21.9)	38 (19.3)	31 (26.3)		
Smoking				0.003	0.2
Never	210 (47.4)	111 (42.9)	99 (53.8)		
Former	190 (42.9)	128 (49.4)	62 (33.7)		
Current	43 (9.7)	20 (7.7)	23 (12.5)		
COPD	31 (7.5)	15 (6.0)	16 (9.6)	0.249	6.5
Diabetes	12 (2.9)	9 (3.6)	3 (1.8)	0.427	6.5
Peripheral artery disease	7 (1.7)	4 (1.6)	3 (1.8)	1.000	6.5
Coagulopathy	61 (13.7)	44 (16.9)	17 (9.2)	0.029	0
Risk factor for VTE	38 (8.6)	15 (5.8)	23 (12.6)	0.019	0.7
History of VTE	354 (79.9)	219 (84.6)	135 (73.4)	0.006	0.2
WHO class				0.006	1.8
I−II	46 (10.6)	32 (12.5)	14 (7.8)		
III	316 (72.5)	192 (75.0)	124 (68.9)		
IV	74 (17.0)	32 (12.5)	42 (23.3)		
Poor mobility	6 (1.4)	3 (1.2)	3 (1.8)	0.943	6.5
Six-minute walk test distance (m), mean (SD)	356.8 (133.2)	379.7 (134.4)	326.3 (125.7)	0.001	33.3
Home oxygen therapy	61 (14.5)	27 (10.9)	34 (19.7)	0.018	5.4
PDEi treatment	76 (17.4)	36 (14.2)	40 (21.9)	0.052	1.8
Mean PAP (mmHg), mean (SD)	46.9 (10.8)	46.0 (10.3)	48.3 (11.2)	0.024	0.9
Cardiac index (l/min/m^2^), mean (SD)	2.1 (0.5)	2.0 (0.5)	2.1 (0.6)	0.195	11.3
PCWP (mmHg), mean (SD)	10.3 (3.6)	10.3 (3.4)	10.2 (3.9)	0.678	16.0
PVR (dynes⋅s⋅cm^−5^), mean (SD)	810.0 (404.2)	763.5 (406.4)	874.6 (393.2)	0.006	6.8
Endarterectomy reported as complete	370 (83.3)	221 (85.0)	149 (81.0)	0.322	0
Year of surgery				0.749	0
1992–2003	72 (16.2)	45 (17.3)	27 (14.7)		
2004–2011	164 (36.9)	94 (36.2)	70 (38.0)		
2012–2020	208 (46.8)	121 (46.5)	87 (47.3)		

Numbers are n (%) unless otherwise noted. VTE: venous thromboembolism; SD: standard deviation; COPD: chronic obstructive pulmonary disease; PDEi: phosphodiesterase inhibitors; PAP: pulmonary artery pressure; PCWP: pulmonary capillary wedge pressure; PVR: pulmonary vascular resistance.

Relative survival was defined as the observed survival divided by the expected survival and was used as an estimate of cause-specific mortality without the need for explicit information on the cause of death. Expected survival in a general population in Denmark matched by age, sex, and year of surgery was obtained from the Human Mortality Database (www.mortality.org). Expected and observed survival curves were constructed with the *strs* Stata command using the Ederer II method.^20^ Data management and statistical analyses were performed using Stata 17.0 (StataCorp LP, College Station, TX, USA) and R version 4.1.0 (R Foundation for Statistical Computing, Vienna, Austria) with the WeightIt^21^ package.

### Missing data

Variables with missing data are shown in Table 1. There were no missing outcome data. In the weighted analyses, missing data were handled by constructing the weights so that the rates of missingness were balanced between the groups (Supplemental Fig. 4).^21^

## Results

A total of 444 patients were included (Supplemental Figs. 1 and 2). There were 260 (59%) men and 184 (41%) women, with a mean age of 61.8 (SD 12.2) and 59.2 (SD 14.2) years, respectively. Although the proportion of women was relatively stable during the study period, there were a few large yearly differences arising from low numbers of operations in these years (Supplemental Fig. 3). Before weighting, there were differences in baseline characteristics between men and women (Table 1). Women were younger, were more often active smokers, and had more risk factors for venous thromboembolism. Coagulopathy was less frequent in women, and fewer had a history of venous thromboembolism. Women were more symptomatic at the time of surgery and had more home oxygen therapy. Hemodynamic parameters such as pulmonary artery pressure, cardiac index, and pulmonary vascular resistance were relatively similar in men and women. After inverse probability of treatment weighting, men and women were well-balanced across all baseline characteristics; all standardized mean differences were <0.1 (Supplemental Table 1 and Supplemental Fig. 4).

### Early mortality

The unadjusted 30-day all-cause mortality was 4.2% (11/260) in men and 9.8% (18/184) in women (p = 0.020). After weighting, the 30-day mortality was 4.6% in men versus 12% in women (p = 0.047).

### Overall survival

The mean and maximum follow-up times were 6.7 (SD 6.2) and 25.9 years, respectively. Before weighting, there was no significant difference in Kaplan–Meier estimated survival between men and women (Supplemental Fig. 5).

**Fig. 1.**
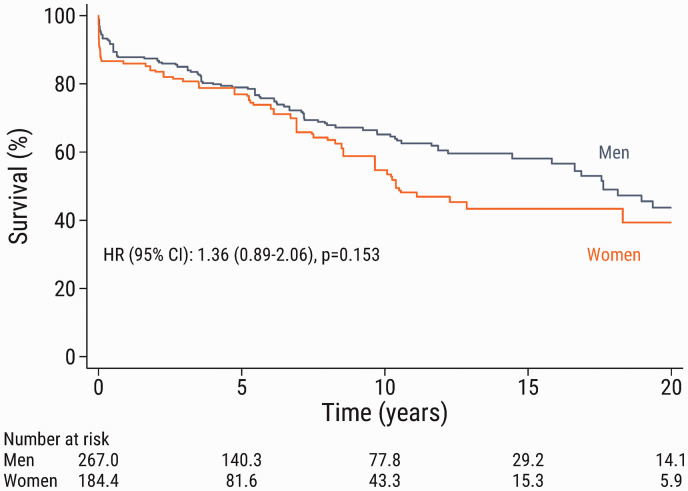
Kaplan–Meier estimated survival according to sex after pulmonary endarterectomy in the inverse probability of treatment weighted population. Note that the numbers of patients in the groups are not necessarily integers because of the inverse probability of treatment weighting. HR: hazard ratio; CI: confidence interval.

The Kaplan–Meier estimated survival in the inverse probability of treatment weighted population is shown according to sex in Fig. 1. There was no significant difference in long-term survival between men and women (HR: 1.36; 95% CI: 0.89–2.06; p = 0.153).

The 1-, 5-, 10-, 15-, and 20-year sex-specific survival (95% CI) in the weighted population and absolute survival differences are shown in Table 2. Men had better survival than women at all time points; however, the difference was not significant.

**Table 2.** Survival in the total study population and according to sex, and the absolute survival difference between men and women after pulmonary endarterectomy.

Time	Total population	Men	Women	Survival difference
Overall survival				
1 year	88 (83–93)	89 (85–94)	86 (80–92)	−3.6 (−8.6–1.5)
5 years	77 (72–82)	79 (73–86)	73 (66–81)	−6.3 (−15–2.4)
10 years	62 (56–69)	65 (58–74)	56 (47–67)	−9.2 (−22–3.5)
15 years	49 (42–57)	53 (44–64)	42 (33–55)	−11 (−25–3.9)
20 years	39 (31–50)	43 (32–57)	32 (21–47)	−11 (−26–4.0)
Conditional on 30−day survival				
1 year	96 (93–99)	96 (92–99)	95 (92–98)	−0.4 (−2.4–1.6)
5 years	85 (80–89)	85 (79–91)	83 (78–89)	−1.4 (−8.2–5.3)
10 years	67 (61–75)	68 (60–77)	65 (56–76)	−2.7 (−15–10)
15 years	53 (45–62)	54 (44–65)	51 (39–65)	−3.3 (−19–12)
20 years	43 (34–54)	43 (33–58)	40 (28–57)	−3.6 (−20–13)

Data are shown as % and (95% confidence intervals) estimated from a flexible parametric survival model after inverse probability of treatment weighting.

### Long-term survival conditional on survival beyond 30 days from surgery

The sex-specific survival conditional on 30-day survival in the weighted population and the absolute survival difference (95% CI) are shown in Table 2. Among patients who survived surgery and the early postoperative period, there were very small differences between men and women.

### Relative survival

The relative survival at 1, 5, 10, 15, and 20 years in the total population and conditional on 30-day survival according to sex are shown in Table 3. In men, the relative survival in the total population ranged from 86% to 92% during 15 years of follow-up. In women, the relative survival was 67% (95% CI: 53%–80%) at 15 years after surgery. In patients who survived beyond 30 days of surgery, the relative survival ranged from 96% to 99% and was equally good in men and women up to 5 years of follow-up. The relative survival at 5 and 10 years remained fairly stable in men at 90% and 94%, respectively, but declined in women at 84% and 75%, respectively. At 20 years, the relative survival was similar in men and women; however, due to a small number of patients at this time point, interpretation must be made with caution.

**Table 3.** Relative survival in men and women after pulmonary endarterectomy.

Time	Number of patients	Men % (95% CI)	Number of patients	Women % (95% CI)
Overall survival				
1 year	199	92 (87–95)	136	89 (83–93)
5 years	137	90 (83–95)	92	86 (79–92)
10 years	76	86 (76–95)	52	75 (64–85)
15 years	29	90 (75–102)	19	67 (53–80)
20 years	11	74 (48–100)	10	71 (52–88)
Conditional on 30−day survival				
1 year	199	96 (92–99)	136	99 (95–100)
5 years	137	94 (88–99)	92	96 (89–100)
10 years	76	90 (80–99)	52	84 (72–93)
15 years	29	94 (79–107)	19	75 (59–88)
20 years	11	77 (50–104)	10	79 (57–97)

CI: confidence interval. The observed survival in the study population was compared with the expected survival in a general population matched by age, sex, and year of surgery. A relative survival of 100% suggests that patients with chronic thromboembolic pulmonary hypertension who underwent pulmonary endarterectomy had the same survival as people of the same age and sex in the general population.

**Fig. 2.**
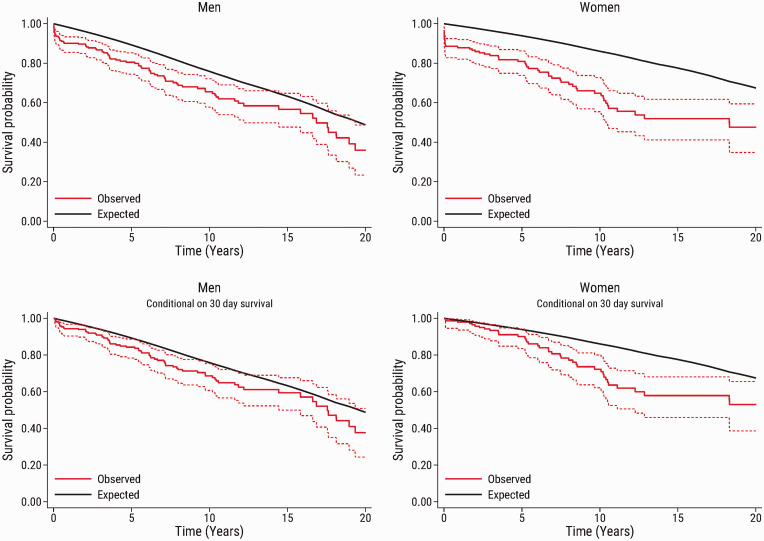
Observed survival (95% confidence interval) in men and women after pulmonary endarterectomy (red solid line and red dashed lines) compared with the expected survival in an age-, sex-, and calendar year-matched Danish population (black line). The upper panel shows the survival in the total study population (n = 444), and the bottom panel shows the survival conditional on patient survival beyond 30 days after pulmonary endarterectomy (n = 415).

**Fig. 3.**
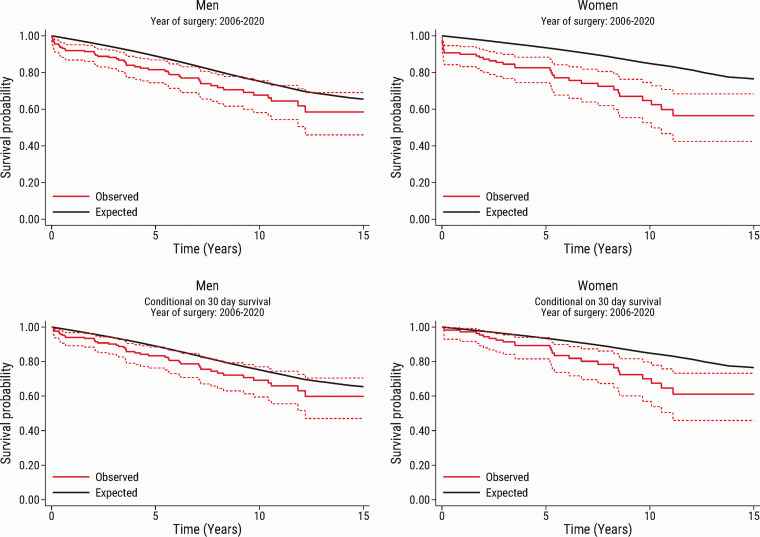
Observed survival in the subset of patients who underwent surgery from 2006 to 2020. The graphs show the observed survival (95% confidence interval) in men and women after pulmonary endarterectomy (red solid line and red dashed lines) compared with the expected survival in an age-, sex-, and calendar year-matched Danish population (black line). The upper panel shows the survival in the total study population (n=346), and the bottom panel shows the survival conditional on patient survival beyond 30 days after pulmonary endarterectomy (n=332).

The observed survival in the total population, and conditional on 30-day survival according to sex, together with the expected survival in an age-, sex-, and year of surgery-matched general population are shown in Fig. 2. In both men and women, the observed survival was lower than the expected survival in the matched general population. In men, the observed survival was close to the expected survival in the matched general population. In women, the difference between the observed survival and the expected survival in the matched general population was more pronounced. Early mortality was higher in women, but even in analyses restricted to women who survived beyond 30 days of surgery, there was a clear difference between the observed survival and the expected survival after more than 5 years of follow-up. These findings were largely confirmed in the subset of patients who underwent surgery from 2006 to 2020 (Fig. 3).

## Discussion

The main finding of the present study was that among patients who underwent PEA for CTEPH, the crude early mortality was higher in women compared with men, but after adjustment for differences in baseline characteristics, the long-term survival did not differ significantly between men and women. Nevertheless, the analyses of relative survival suggested that the observed survival in men was close to the expected survival in the matched general population, while the survival in women deviated to a larger extent from the matched general population. These findings suggest that there are sex-specific differences in prognosis following PEA for CTEPH.

Barco et al.^11^ suggested better five-year survival in women compared to men, even though women underwent PEA less often. Their prospective CTEPH registry included 679 patients, of whom 339 were women (50%). Of the 339 women, 183 (54%) underwent PEA, compared with 221 of the 340 men (65%). In the group of non-surgically treated patients, women had longer median diagnostic delay than men, while women who underwent PEA had shorter diagnostic delay than men. In the non-operated group, the presence of microvascular disease was more common in women (19.5%) than in men (13.5%) and was the most important reason for not undergoing PEA. More women had high pulmonary vascular resistance (PVR) or old age as contraindications to surgery than men. Men who underwent PEA were more often smokers and had a history of coronary artery disease, while women had more thyroid disease and were more often obese. The proportion of women undergoing PEA was higher in high-volume centers compared with low-volume centers. At one-year postoperatively, all-cause mortality was 5.5% in women and 6.8% in men. At long-term follow-up after PEA, cardiovascular mortality was 4.9% in women and 8.6% in men. The annual death rate was 2.7% in women and 4.8% in men. According to the authors, the cohort was too small to perform sex- and PEA-stratified survival analyses.

Acute pulmonary embolism (PE) is considered one of the major risk factors for development of CTEPH; approximately 70% of patients with CTEPH had prior venous thromboembolism in a previous study.^22^ Sex differences have been investigated in Denmark, Sweden, and the United States, with findings that female sex was slightly more common (52%–53%) in cohorts of studies on the incidence of acute PE.^23–25^ Outcomes after acute PE seemed to be similar between the sexes after adjustment for age and comorbidities, but the clinical presentations differed between men and women.^25–27^ In some studies on acute PE, the thrombotic burden was higher in women, and women had more right heart dysfunction and bleeding complications due to treatment, but also better survival than men.^28^ Pribish et al.^25^ evaluated an American cohort and found that women with acute PE were more likely to have normal right ventricular (RV) size than men on echocardiography.

In Japan, there is a special phenotype of CTEPH associated with HLA-B*5021. There is also an overall 2:1 female predominance for CTEPH. Shigeta et al.^29^ investigated a cohort of 150 patients to characterize the female phenotype of CTEPH in Japan. Almost half of the female patients were positive for HLA-B*5021. Women had better RV function, lower right atrial pressure, and better cardiac index than men. Women less frequently had prior venous thromboembolism and had worse PaO_2_. There was no significant difference in survival between the sexes after PEA, even though women had less reduction in PVR postoperatively than men.

Two studies have investigated sex differences in hemodynamic reactions in CTEPH.^30,31^ Yang et al.^30^ investigated possible sex differences in hemodynamics using acute vasoreactivity testing in CTEPH to predict outcomes. Acute vasoreactivity testing was used as a measure for compliance of the pulmonary vascular bed. They found that both sexes had unique hemodynamic responses and that these parameters could independently predict event-free survival. Chen et al.^31^ examined sex-specific cardiopulmonary exercise testing in inoperable CTEPH and found that men and women had different predictors for PVR. In men, the nadir minute ventilation/cardon dioxide output was an independent predictor for PVR. In women, the predictor for PVR was oxygen uptake efficiency plateau. The same group performed a similar study in patients with idiopathic pulmonary arterial hypertension and found sex-specific differences in predictors for PVR and cardiac output.^32^

A study by Swift et al.^33^ investigated differences in the RV between men and women with idiopathic pulmonary arterial hypertension using magnetic resonance imaging. Men with idiopathic pulmonary arterial hypertension had proportionally lower RV ejection fraction, RV stroke volume, and left ventricular (LV) stroke volume. Estimated RV mass, mean pulmonary artery pressure, and PVR were similar between the sexes on magnetic resonance imaging. The authors hypothesized that adaptive remodeling of the RV in response to increased afterload in idiopathic pulmonary arterial hypertension is more effective in women.^33^

A possible explanation for the present findings could be that women have a more adaptive RV and therefore objective signs of disease were visible later in the disease course, resulting in relatively worse outcomes for women due to the late diagnosis. Prior studies identified that high PVR as a negative prognostic factor for survival in CTEPH and after PEA.^6,34,35^ In our study, men had more known coagulopathy and more history of venous thromboembolism, thus making it easier for clinicians to consider CTEPH and possibly arrive at a correct diagnosis earlier. While we found no strong evidence for more severe hemodynamics in women versus men preoperatively, there may still be sex-specific differences in the timing of surgery. Our results showed that women were more symptomatic, were more often on phosphodiesterase inhibitors, and had more home oxygen treatment at the time of PEA compared with men. We found no evidence for inferior surgical results for women to explain the higher crude early mortality in women.

Future studies related to sex differences in CTEPH and PEA are warranted to confirm our findings and deepen our understanding with the goal of improving the prognosis in women.^8^ Several questions remain. For example, whether it is harder to establish a correct diagnosis of CTEPH in women compared with men, whether women undergo PEA at a later CTEPH disease stage compared with men, and if so, whether this affects prognosis and survival, and whether the phenomenon is related to delays from the patients or doctors.

### Study limitations

The present results could have been affected by changes in diagnosis, referral, and care of patients with CTEPH during the study period of almost 30 years. All PEA patients who underwent surgery in Stockholm and Aarhus were included in the study, but no information on non-surgically-treated CTEPH patients was available; thus, patient selection may have affected the results. The lack of information regarding some comorbidities or other unknown factors that possibly influence morbidity and mortality may also have affected the results of the study. Another limitation of the study was the lack of information on anticoagulation strategy, post-PEA treatments, such as targeted medication or balloon pulmonary angioplasty that may have influenced the long-term prognosis. We also lacked information regarding the time-interval between diagnosis and surgery, which may differ in men and women, and could have influenced prognosis.

## Conclusions

This study suggests that there are sex-specific differences in prognosis following PEA for CTEPH. In patients who underwent PEA for CTEPH, the crude early mortality was higher in women compared with men, but after adjustment for differences in baseline characteristics, the long-term survival did not differ significantly between men and women. However, the analyses of relative survival suggested that the observed survival in men was close to the expected survival in the matched general population, while the survival in women deviated to a larger extent from the matched general population.

## Supplemental Material

Supplemental_Material_anon.docx - Supplemental material for Sex and survival following pulmonary endarterectomy for chronic thromboembolic pulmonary hypertension: a Scandinavian observational cohort study Click here for additional data file. Supplemental material, sj-pdf-1-pul-10.1177_20458940211056014 for Sex and survival following pulmonary endarterectomy for chronic thromboembolic pulmonary hypertension: a Scandinavian observational cohort study by Janica Kallonen, Kasper Korsholm, Fredrik Bredin, Matthias Corbascio, Mads Jønsson Andersen, Lars Bo Ilkjær, Søren Mellemkjær and Ulrik Sartipy in Pulmonary Circulation
