# Supplementary material for: Sex and survival following pulmonary endarterectomy for chronic thromboembolic pulmonary hypertension: a Scandinavian observational cohort study
Source: Pulm Circ. 2021 Oct 27;11(4):20458940211056014. doi: 10.1177/20458940211056014 (PMC8673940; doi:10.1177/20458940211056014)

**Supplemental Material**

**Sex and survival following pulmonary endarterectomy**

**for chronic thromboembolic pulmonary hypertension:**

**a Scandinavian observational cohort study**

***Running head: Sex−specific survival after surgery for CTEPH***

Contents

[Supplemental Table 1 3](#_Toc73438611)

[Supplemental Figure 1 5](#_Toc73438612)

[Supplemental Figure 2 6](#_Toc73438613)

[Supplemental Figure 3 7](#_Toc73438614)

[Supplemental Figure 4 8](#_Toc73438615)

[Supplemental Figure 5 9](#_Toc73438616)

| Supplemental Table 1**.** Baseline characteristics in patients who underwent pulmonary endarterectomy in Sweden and Denmark between 1992 and 2020 after inverse probability of treatment weighting. | | | |
| --- | --- | --- | --- |
| Variable | Men* | Women* | SMD |
| Number of patients | 267.0 | 184.4 |  |
| Center |  |  | 0.014 |
| Denmark | 194.0 (72.7) | 136.7 (74.1) |  |
| Sweden | 72.9 (27.3) | 47.8 (25.9) |  |
| Age (years), mean (SD) | 61.9 (11.8) | 61.1 (13.9) | 0.058 |
| Body mass index (kg/m^2^) |  |  |  |
| <18.5 | 1.3 (0.7) | 1.5 (1.1) | 0.005 |
| 18.5–24.99 | 83.5 (42.8) | 55.0 (42.0) | 0.008 |
| 25–29.9 | 69.2 (35.5) | 44.5 (34.0) | 0.015 |
| ≥30 | 41.0 (21.0) | 29.9 (22.8) | 0.018 |
| Smoking |  |  |  |
| Never | 112.7 (42.3) | 80.4 (43.6) | 0.013 |
| Former | 123.6 (46.4) | 84.2 (45.7) | 0.007 |
| Current | 30.0 (11.3) | 19.9 (10.8) | 0.005 |
| COPD | 23.1 (9.3) | 15.5 (9.1) | 0.002 |
| Diabetes | 7.3 (2.9) | 2.8 (1.7) | 0.012 |
| Peripheral artery disease | 3.0 (1.2) | 2.1 (1.2) | 0.000 |
| Coagulopathy | 34.8 (13.0) | 22.1 (12.0) | 0.010 |
| Risk factor for VTE | 21.3 (8.0) | 17.3 (9.5) | 0.014 |
| History of VTE | 217.9 (81.8) | 146.9 (79.7) | 0.021 |
| WHO class |  |  |  |
| I−II | 28.3 (10.8) | 17.2 (9.6) | 0.013 |
| III | 197.5 (75.6) | 133.9 (74.3) | 0.014 |
| IV | 35.3 (13.5) | 29.1 (16.1) | 0.026 |
| Poor mobility | 3.0 (1.2) | 2.3 (1.3) | 0.001 |
| 6-min walk test distance, quartiles |  |  |  |
| Q1 (low) | 46.6 (25.6) | 35.0 (27.9) | 0.023 |
| Q2 | 47.9 (26.3) | 33.6 (26.8) | 0.005 |
| Q3 | 43.3 (23.8) | 30.0 (24.0) | 0.001 |
| Q4 (high) | 44.1 (24.2) | 26.7 (21.3) | 0.029 |
| Home oxygen therapy | 34.3 (13.7) | 25.6 (14.7) | 0.011 |
| PDEi treatment | 39.4 (15.0) | 31.1 (17.0) | 0.020 |
| Mean PAP (mmHg), mean (SD) | 46.2 (10.4) | 46.5 (11.4) | 0.032 |
| Cardiac index (l/min/m^2^), mean (SD) | 2.1 (0.53) | 2.1 (0.51) | 0.033 |
| PCWP (mmHg), mean (SD) | 10.2 (3.4) | 10.2 (3.5) | 0.012 |
| PVR (dynes•s•cm^-5^), mean (SD) | 807 (559) | 820 (369) | 0.032 |
| Endarterectomy reported as complete | 227.0 (85.0) | 154.1 (83.6) | 0.015 |
| Year of surgery |  |  |  |
| 1992−2003 | 43.3 (16.2) | 27.8 (15.0) | 0.012 |
| 2004−2011 | 96.3 (36.1) | 69.7 (37.8) | 0.017 |
| 2012−2020 | 127.3 (47.7) | 87.0 (47.2) | 0.005 |
| Numbers are n (%) unless otherwise noted.  *The overall numbers of patients in each group are not necessarily integers owing to inverse probability of treatment weighting.  SMD = standardized mean difference, SD = standard deviation, COPD = chronic obstructive pulmonary disease, PDEi = phosphodiesterase inhibitors, PAP = pulmonary artery pressure, PCWP = pulmonary capillary wedge pressure, PVR = pulmonary vascular resistance, VTE = venous thromboembolism | | | |

Supplemental Figure 1. Number of pulmonary endarterectomies per year.


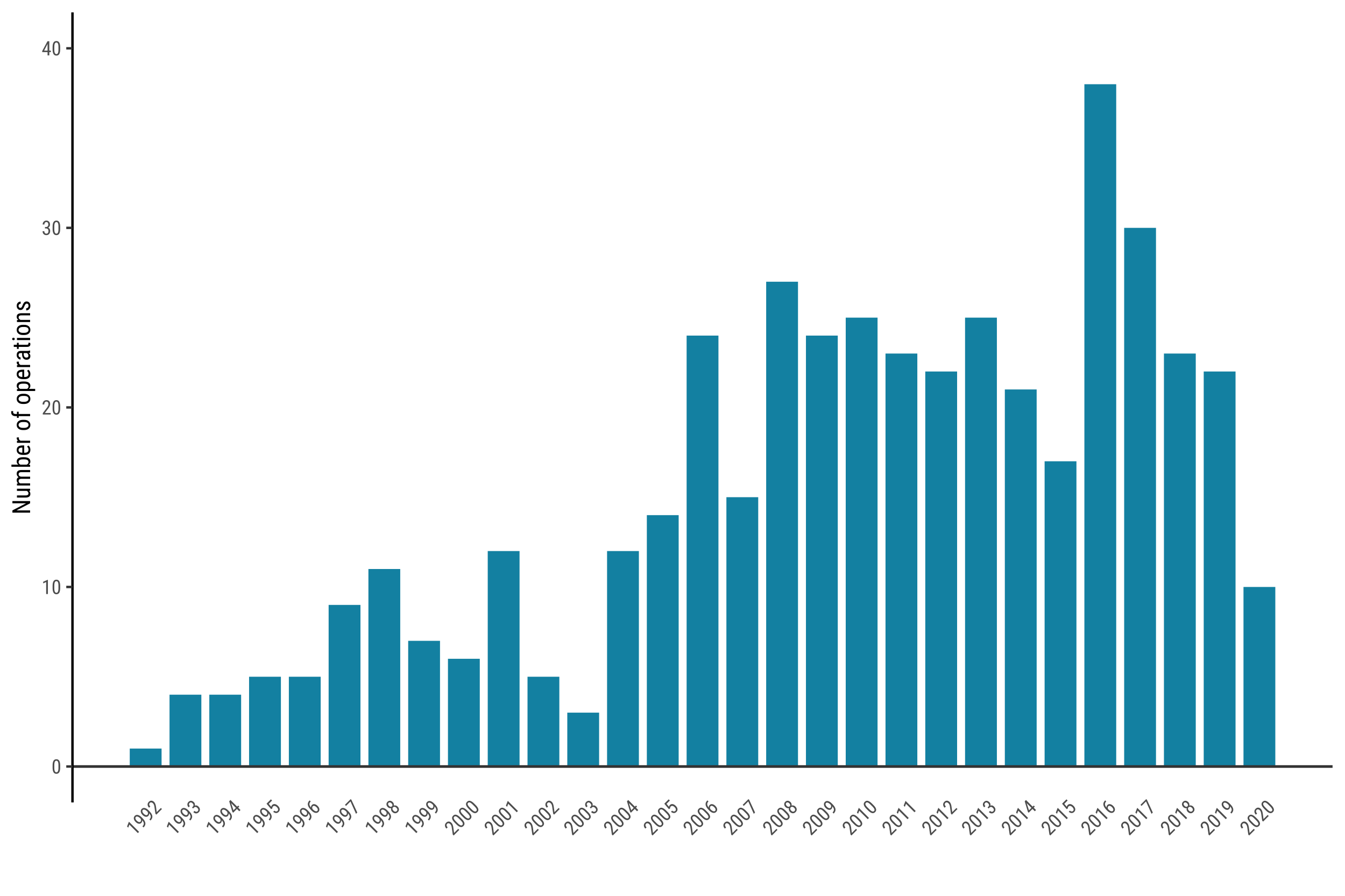


Supplemental Figure 2. Number of pulmonary endarterectomies per year and center.


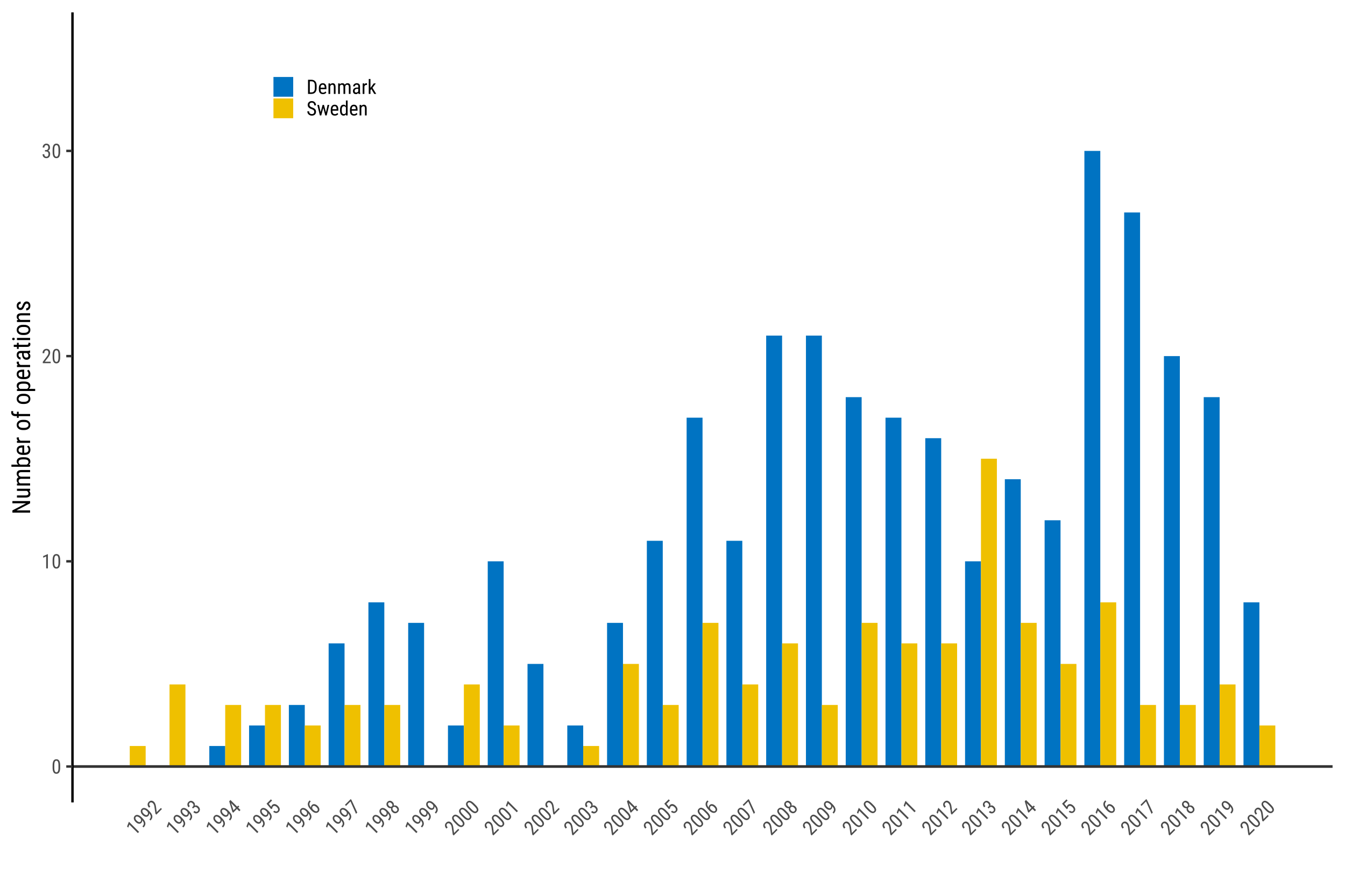


Supplemental Figure 3. The yearly proportion of women who underwent pulmonary endarterectomy in the total study population (n = 444).


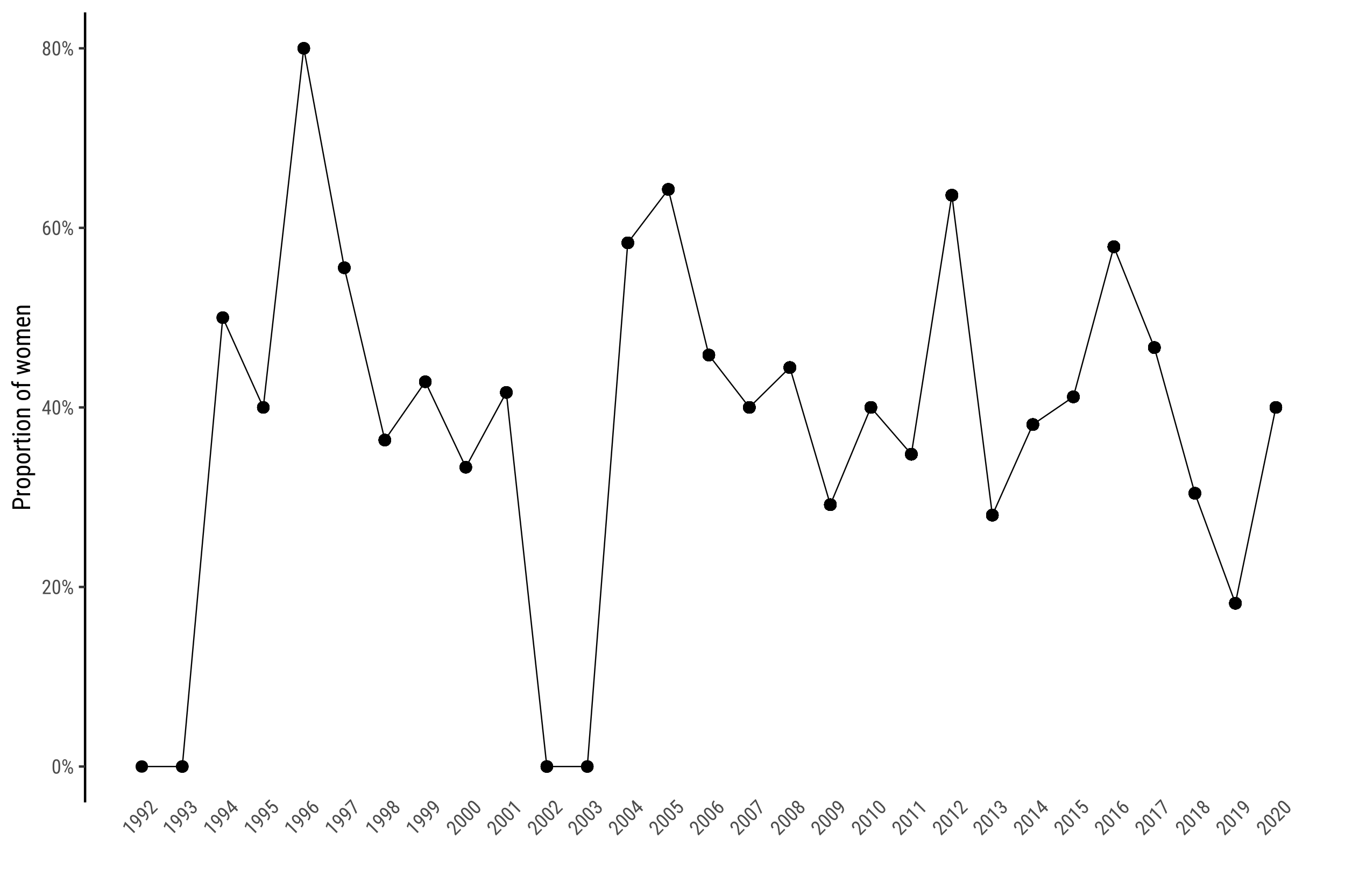


Supplemental Figure 4. Absolute standardized differences before (hollow circles) and after (filled circles) inverse probability of treatment weighting.


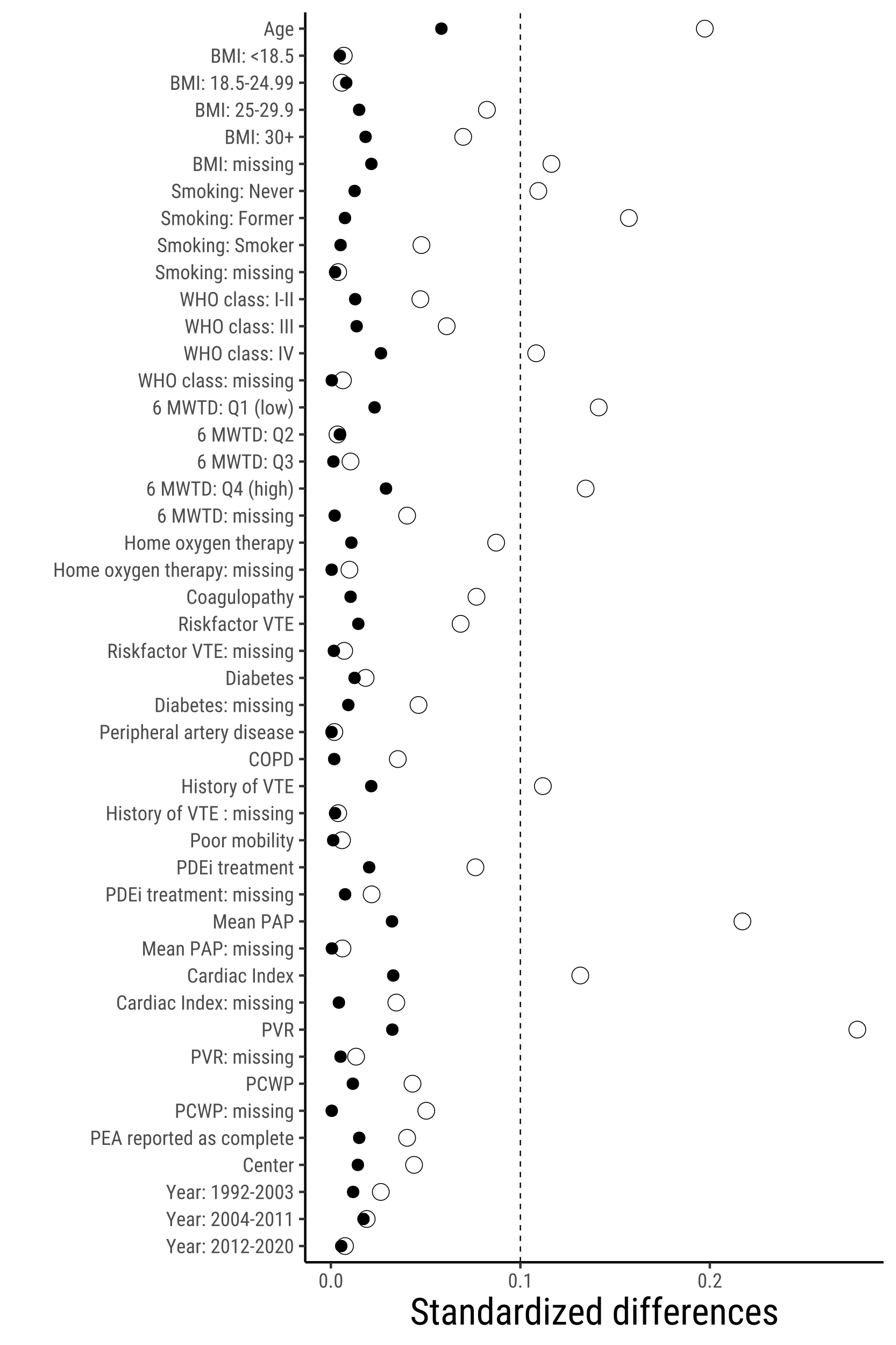


Supplemental Figure 5. Kaplan-Meier estimated survival according to sex after pulmonary endarterectomy before weighting in the total study population (n = 444).

*HR = hazard ratio, CI = confidence interval*


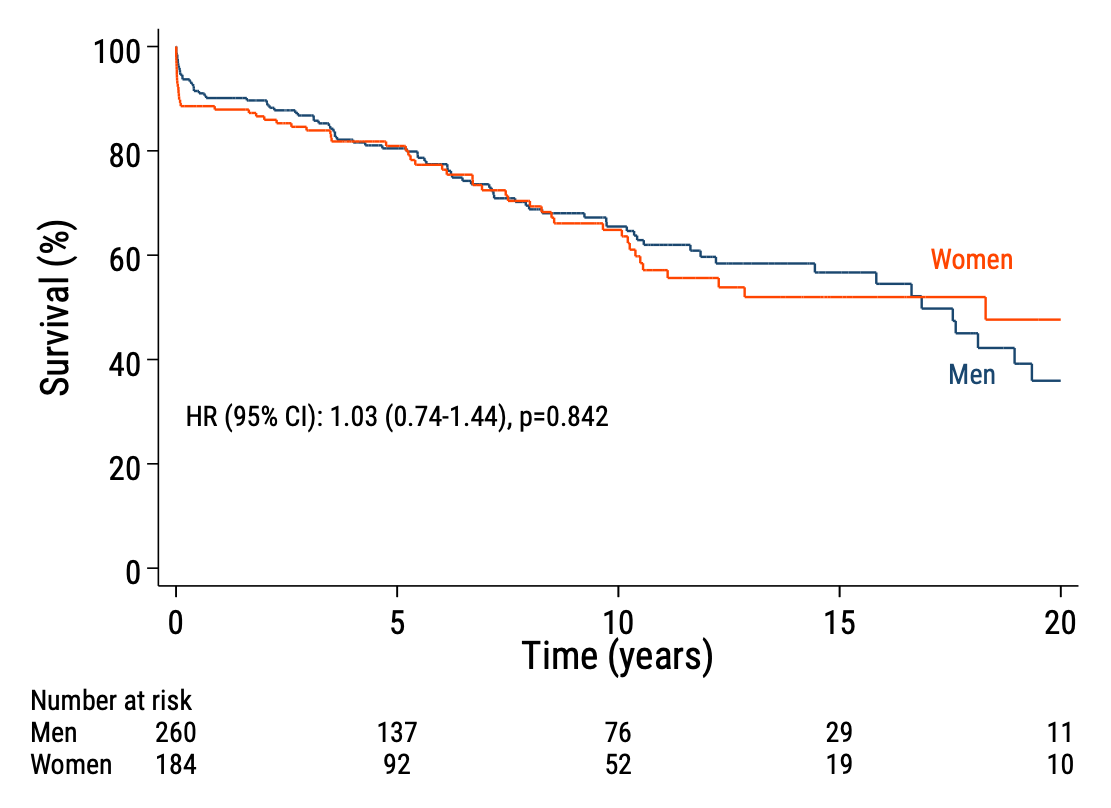

Supplement: Supplemental_Material_anon.docx - Supplemental material for Sex and survival following pulmonary endarterectomy for chronic thromboembolic pulmonary hypertension: a Scandinavian observational cohort study [file Supplemental_Material_anon.docx]
